# Managing change in the nursing handover from traditional to bedside handover – a case study from Mauritius

**DOI:** 10.1186/1472-6955-4-1

**Published:** 2005-01-28

**Authors:** Hemant K Kassean, Zaheda B Jagoo

**Affiliations:** 1 Department of Health and Medical Sciences and Management, University of Mauritius, Reduit, Mauritius; 2 Department of Health and Medical Sciences, University of Mauritius, Reduit, Mauritius

## Abstract

**Background:**

The shift handover forms an important part of the communication process that takes place twice within the nurses' working day in the gynaecological ward. This paper addresses the topic of implementing a new system of bedside handover, which puts patients central to the whole process of managing care and also addresses some of the shortcomings of the traditional handover system.

**Methods:**

A force field analysis in terms of the driving forces had shown that there was dissatisfaction with the traditional method of handover which had led to an increase in the number of critical incidents and complaints from patients, relatives and doctors. The restraining forces identified were a fear of accountability, lack of confidence and that this change would lead to more work. A 3 – step planned change model consisting of unfreezing, moving and refreezing was used to guide us through the change process. Resistance to change was managed by creating a climate of open communication where stakeholders were allowed to voice opinions, share concerns, insights, and ideas thereby actively participating in decision making.

**Results:**

An evaluation had shown that this process was successfully implemented to the satisfaction of patients, and staff in general.

**Conclusion:**

This successful change should encourage other nurses to become more proactive in identifying areas for change management in order to improve our health care system.

## Background

This study was undertaken in a 28 – bedded gynaecological ward catering for female patients aged 16 and above. There were 21 nurses based in this ward of whom 14 were qualified and the remaining were health care assistants, with experience ranging from 1 ^1/2 ^– 33 years. The shift handover in this ward was conducted as "a ritual inheritance," [3] distant from patients hearing and vision, such as the ward manager's office or the nurses' station, thus excluding patients participation in their care. The traditional handover used to consist of one-way communication, where the nurse in charge gave the relevant information and instructions to the nurses resuming their shift. A very salient feature of the handover was the absence of individual care planning and where all information about patients was either written in the ward diaries or in the patient files or nursing notes. The sample size of patients involved in the evaluation part of the study was 58.

The verbal handover was derived from written information on the office white board which included the patient's name, bed number, medical diagnosis and the treating doctor. This was in line with the findings of Sherlock [12] who argued that the shift handover was characterized by a focus on the biomedical model and marginalized the psychosocial aspects of care. The same style of reporting was repeated from one shift to another. As a result, the contents would sometimes degenerate into irrelevant and outdated statements, unrelated to the patient progress and often judgmental in nature with the likelihood of leading to omissions in care. It was therefore not uncommon that nurses were questioned on their practice by the ward manager or the treating doctor which gave rise to a blaming culture among nurses. There was also a level of dissatisfaction among patients who felt that they were not being involved enough in their care.

### Diagnose need for change

The root cause of the problem identified was the model of handover used to communicate clinical information. As a benchmark, the findings from evidence on the bedside handover were used to give meaning and strength to the proposed change. Bedside handovers offer an immediate solution to the many problems that are associated with the traditional handover [5,15,16]. It has further advocated that bedside handover lay more emphasis on individualized patients care whereas bedside reporting is the most frequently used model of handover [5]. It puts the patients central to all care activities and does not rely purely on verbal information but which combines the key principle of patients/clients involvement and participation. In the same context, patients involved in handovers gain access to information that is thought to provide them with comfort and speed recovery [10]. Bed-side reporting makes it possible for nurses starting their shift to obtain a better insight into the care each patient requires [6]. Patients can discuss their health by asking questions and it was found to improve the consistency and continuity of patients care. The information style of bedside handover was informative, personal, shorter and comprehensive. In the light of the above findings, bedside handover had become a valid option for change in this ward.

## Methods

### Theories underpinning the change process

An adaptation of Spradley's 8-step model and Lewin's 3-step model of *Unfreezing*, *Moving *and *Refreezing *provided us with useful frameworks for our change management [9,14].

### Unfreezing

Unfreezing is about encouraging people think about the current situation and helping them recognise the need for change [5]. Change to be initiated requires a sense of direction and considerable power of leadership [8]. The authors were also guided by the work of Swansburg and Swansburg, [15] who argued that "transformational leaders are seen in health care organizations as a commitment to excellence."

The first move therefore was to create awareness by communicating the proposed change to all those who were going to be affected by the new practice: the nurses, patients and the ward manager so that they all had a shared vision of an improved handover system. A goal-seeking behavior with a clear logical sequence of action, were demonstrated throughout the process as advocated by Lancaster and Lancaster [8,17]. Research based articles were also used to demonstrate how this system was successfully implemented in different areas of the health care system.

The proposed change was announced in advance by using different communication channels, e.g. personal contact with individual nurses, staff information/notice board by the authors. This initiated informal discussion among nurses of the ward by creating a cognitive dissonance which led to a quest for more information about the new handover. This consultation phase allowed the nurses to discuss various clinical scenarios and analyse the constraints and benefits of the new proposal in the local context. They were also involved in group work to identify and make proposals on how to deal with some of the problems that we may encounter in our local context e.g. handover coinciding with ward rounds or emergency situations and patients too distressed to talk. Case studies and research articles on this topic were used for discussion and to further reinforce the beliefs of staff of the ward that the current practice had shortcomings and could be improved. The status quo was therefore unsettled and this enabled us to rule out the first resistance through a normative re-educative strategy. A group of senior nurses who had experience in this particular area agreed take turn to act as mentors in order to facilitate this process and offer support to their junior colleagues in the first week until they become confident to carry out the process without supervision.

### Analysing the alternative options

The extensive literature search also provided us with options for alternatives to bedside handover. These were thoroughly debated before reaching a decision. The options considered were the following:

1) Tape recorded handover

2) Computer generated handover using information technology

3) Bedside handover, based on individualized care plan

The 'SMART' criteria were used to evaluate the feasibility of the alternatives to bedside of handover. The tape recorded handover would require a tape recorder being taken around to each of the patients and the interaction recorded. An informal discussion with the patients revealed that this method was distractive and the majority of them did not feel comfortable about their conversation being recorded. With regards to the computer generated handover using information technology, the patients felt this system will not enable them to engage fully in the process. It was also felt that since the first two options required extra financial, technical resources for implementation, these would not be feasible in the first instance whereas the bedside handover gained unanimous support from both patients and staff. This was also more realistic in term of its applicability in our practice area. It was specific, measurable in terms of its performance and achievable within existing resources and a defined time frame. Its foundation rested on evidence base practice, which showed theoretical soundness.

### Selecting the change

There was a shared vision about the worth of the proposed change by the team and consequently bedside handover was logically considered as the best option for change. The vision formulated was that in three months' time, bedside handover would become the normal shift handover process of the ward. The mission statement agreed was "all handovers would be carried out at the patients' bedside between the incoming and outgoing nursing staff with the patients' involvement."

### Force-field analysis

A force field analysis, as shown in table 1 was carried out to evaluate the driving and the restraining forces for the change as per Lewin's model [9]. The driving forces resided in the support of the ward manager, peers, evidence based arguments and our determination to see the change happen. The restraining forces were mostly related to a lack of information and uncertainty surrounding the change process. Other significant issues that were identified to cause resistance to the change were lateness at work, non-overlapping of shifts and maintaining confidentiality of patient's information.

**Table 1:** A force field analysis using Lewin's (1951) driving and restraining forces

**DRIVING FORCES**	**RESTRAINING FORCES**
• Critical incidents on the increase • Care given predominantly biomedical in orientation • Complaints from patients, doctors and relatives on the rise. • Increase in discharge against medical advice • Staff knowledgeable in change management • Ward manager's and peer lending support • Familiarity with ward culture	• Ritualism and tradition • Fear that this may lead to more work • Lack of confidence on the part of some nurses • Fear of increased accountability • Problems associated with arriving late at work • Problems associated with disclosure of confidential information

### Planning the change

Careful planning is essential if trauma is to be minimized [2]. It was quite important for us to provide information so as to unlock the status quo. This was done by drafting a protocol, (table 2) on a six points systematic step on how to proceed in practice with the change. This protocol was piloted over 2 morning and 2 evening handover sessions to ensure validity and reliability. There were no changes required to the protocol following the pilot study.

**Table 2:** Results of protocol with 6 criteria based on observational data on 10 handovers

1.	Outgoing and incoming nurses meet in the office to get a report on confidential matters.	100%
2	Outgoing and incoming nurses then move on to the patient's bedside.	100%
3	Nurses introduce themselves to the patient and initiate handover from patient's him/herself in the first instance.	100%
4	Patient's progress is reviewed as per care plan with a discussion of the future care of the patient.	100%
5	Any other queries from patient is dealt with	100%
6	Session with patient is concluded satisfactorily	90%

The time frame earmarked to implement the change was three months starting from the 8^th ^of February 2003 up to 8^th ^May 2003. One-month time was judged sufficient to unfreeze the situation and the remainder to implement and evaluate the change.

### Selecting strategies for change

Choosing a strategy for the change process depended upon various factors and good interpersonal relationship was a critical factor. It has been proposed that strong leadership and excellent communication skills were essential if an atmosphere of trust was to be engendered [7,8]. With this in mind, the change was announced in advance to encourage the nurses. It also offered the opportunity to share the reaction of colleagues where some valuable proposals were proposed, for example, how to cater for lateness at work, non-overlapping of shift as well as dealing with confidentiality of information.

Confidential issues related to matters that the patients brought up during the admission procedure and during their stay, certain issues that were brought up during ward rounds and from the patients own requests.

In cases of occasional lateness in resuming work, the handover would proceed with the other patients in first the instance and if the staff was still late, then some other colleague would step in her place. Reassurance was given with respect to 'no substantial overlapping' of shift in that it would not have major bearing on the handover process by explaining that shorter handover can reduce the likelihood of information overload and result in concise and pertinent information being exchanged as per care plans. There was a general agreement that fifteen minutes as officially allocated for handover would be sufficient for this purpose. Assurance was also given that confidentiality of patients' clinical information would be taken into consideration in drafting a protocol for bedside handover, as shown in table 2.

### Empowering the staff

Several meetings were organized with different groups of nurses to explain and clarify any shortcomings and to reach a consensus. This approach was recommended by Driscol [4], as it empowers the team to make the change for itself, without instruction or oversight and is believed to be a strategy for an effective and lasting transformation in a team spirit. The empirical rational strategy was used to convince others of the veracity of the change by making reference to evidence base documentation on the positive outcomes that bedside handover might bring, for example, increase patient satisfaction. Nurses within the ring of informal leaders were gradually encouraged to take some of the ownership of the change by entrusting role model responsibilities to them. This proved to be quite successful as a strategy to encourage participation to create attitudinal and behavioral change. Eventually, there was more acceptance and collaboration on the part of the team to implement the change. In keeping with Skinner's theory [13], positive reinforcement, was used to praise and encourage staff. The ward manager helped in the reinforcement process by complimenting the whole team for their excellent effort to bring the change during the weekly meeting of staff. The strategy of facilitation also involved providing training in the new skill demanded by the change. Mocked handover exercises were demonstrated with the different steps of bedside handover to different groups of nurses. This was done by adopting a democratic leadership style engendering a participative approach, which in turn generated a degree of enthusiasm for the change.

### Moving stage

Following a pilot handover session involving senior staff in a participant and an observer capacity over 2 morning and 2 evening handover sessions, which did not require any major changes, implementation of the bedside handover was started on 8^th ^of March 2003. For the first week, six senior staff who had experience in this area volunteered and took turn to continue to be present in as many handovers as observers and participants, to monitor and reinforce the established protocol step by step.

They also provided clarification and support to staff in cases of difficulty, and helped evaluate the extent of change that had taken place in an effective manner. The nurses present during the handover had no difficulty in adapting to this new situation, using a care plan incorporating a more psychosocial and patient-centered approach to bedside handover with the patients' participation.

## Results

### Evaluation of the change

The evaluation of the implementation of bedside handover was carried out in two distinct phases. A protocol, as shown in table 2, was developed which included 6 criteria was duly filled after every shift handover. As a benchmark, a good handover was one where at least five of the criteria were strictly followed. The data collection consisted of ten non-participant observation handovers. Semi-structured interviews, using a questionnaire derived from a focus group of staff and patients as shown in table 3, with 40 patients were carried out to get their perceptions of the new handover. This was done randomly, consisting of both morning and evening handovers over a period of a week by a staff specifically chosen for this job from another ward to prevent bias from the hawthorn effects and ensure validity.

**Table 3:** Evaluation of bedside handover from patients' perspectives – Results of semi-structured interviews with 40 patients

1. Do the outgoing and incoming nurse come to your bedside to handover in the morning and in the evening during the change of shifts?	95% – yes
2. How do you feel about their presence at your bedside to discuss your care?	100% – ok and most of them said it was a good thing
3. Do the nurses involve you in your care planning?	80% – yes, 10% – to a certain extent, 10% not sure
4. Are you satisfied with the way information about your care is passed on and followed by the incoming nurses?	100% – no problem
5. How do you feel issues of confidentiality are handled?	100% – sensitively
6. Any other comments you would like to make to improve on shift handovers?	1) Satisfied – 100% Other comments: 2) Doctors and other professionals to adopt this approach 3) Nurses should spend more time talking to them, not during the handover period only 4) Would like this to happen in all wards

Analysis of results of the observational data on 10 handovers, Table 2, showed that the first 5 criteria were met at 100% and the 6^th ^criteria at 90%. In one of the sessions, the nurse had left the patient whilst the bedside handover was in progress to attend to another patient without explaining the reason for this short absence to him which accounts for the 90%.

Analysis of the results of semi-structured interviews with 40 patients, Table 3, show that a 96% overall satisfaction level was achieved. This was beyond our expectations, as we had targeted a success rate to be 80% initially. We had to be cautious about the result for it could be either most of the staff had accepted the change or just doing it in this euphoric phase.

### Refreezing phase

The result was evaluated at a full staff meeting and the ward manager and colleagues recognized the change. Despite unlearning of the old practice had taken place two nurses still displayed some difficulties with the new handover as they were always eager to report everything themselves rather than allowing the patients to have a say. After a reassessment of the situation, accurate feedback was given to them. With the group support, they became used to the new system by observing their colleagues in action during the handover and doing it in turn. After a couple of sessions they became fully conversant with the new system. By this time, this project was ready for the refreezing stage.

## Discussion

One of the major difficulties encountered was to rally everybody behind this project. The normative re-education, in line Bernhard and Walsh [1], was used in order to help nurses value the new knowledge and create a readiness for learning. Various tasks identified for the future, for example on how to deal with issues of confidentiality, patients who would keep talking endlessly making the process drag on for a long time were allocated to members of the team according to their expertise to prepare so that they could be discussed in depth during the next meeting. A flexible and humanistic in approach was adopted in dealing with conflict, and resistance was not underestimated. Suggestions were treated with respect and dignity. Considerable effort was made to maintain good interpersonal relationship and to highlight motivating factors and safety needs. Constructive feedback was provided on their level of performance. Positive behaviors were rewarded equally in terms of recognition and praise and often with a simple and genuine "thank you". Application of this knowledge was reinforced from day one when this new handover became operational into the practice area through continuous coaching, supervision and mentorship.

## Conclusion

Managing change in a hospital set up is a daunting task as it involves a change in the attitude and behavior of staff in a complex environment in order to gain their collaboration. The concept of no pain no gain was very evident throughout the process. Lewin's 3 – stage model was useful in implementing the change in a planned and structured way. Resistance was overcome by creating a climate which encouraged open communication. The support of the ward manager and key stakeholders were significant. Evaluation has shown that the new system of handover is working well but monitoring will be ongoing with evaluation of a larger sample of patients. This change has been an enriching experience for the staff, and has generated enthusiasm and given them confidence to question some of the practices on the ward. This new approach to handover can therefore be implemented in other areas of practice and evaluated to ensure that they are meeting patients' satisfaction. Further studies can be undertaken to explore how the multidisciplinary team could further consolidate this process.

## Competing interests

The author(s) declare that they have no competing interests.

## Authors' contributions

HKK taught this module and supervised this change management project. ZBJ implemented this change in the gynaecological ward as part of her assessment in the "Management Development" module. Both authors wrote this manuscript, read and approved the final version.

## Pre-publication history

The pre-publication history for this paper can be accessed here:


